# Sirt1 Promotes the Restoration of Hepatic Progenitor Cell (HPC)-Mediated Liver Fatty Injury in NAFLD Through Activating the Wnt/β-Catenin Signal Pathway

**DOI:** 10.3389/fnut.2021.791861

**Published:** 2021-12-15

**Authors:** Qinjin Li, Yuqing Gong, Yi Wang, Bingbing Liu, Yi Chu, Sisi Gui, Yazhen Zheng, Xiaodong Chen

**Affiliations:** ^1^Key Laboratory of Agricultural Animal Genetics, Breeding and Reproduction of Ministry of Education, College of Animal Science and Technology & College of Veterinary Medicine, Huazhong Agricultural University, Wuhan, China; ^2^College of Life Science and Technology, Huazhong Agricultural University, Wuhan, China

**Keywords:** NAFLD, Sirt1, hepatic progenitor cells, HFD, liver injury and repair

## Abstract

Non-alcoholic fatty liver disease (NAFLD) has developed into the world's largest chronic epidemic. In NAFLD, hepatic steatosis causes hepatocytes dysfunction and even apoptosis. The liver has a strong restoration or regeneration ability after an injury, however, it is unclear through which pattern fatty liver injury in NAFLD is repaired and what the repair mechanism is. Here, we found that in the high-fat diet (HFD)-induced NAFLD mice model, fatty liver injury caused the significant ductular reaction (DR), which is a marker to promote the repair of liver injury. SOX9^+^ and HNF4α^+^ biphenotype also suggested that hepatic progenitor cells (HPCs) were activated by fatty liver injury in the HFD-elicited NAFLD mice model. Concurrently, fatty liver injury also activated the Wnt/β-catenin signal pathway, which is a necessary process for HPC differentiation into mature hepatocytes. However, Sirt1 knockdown weakened HPC activation and Wnt/β-catenin signal in Sirt1^+/−^ mice with HFD feeding. In rat-derived WB-F344 hepatic stem cell line, Sirt1 overexpression (OE) or Sirt1 activator–Resveratrol promoted HPC differentiation via activating Wnt/β-catenin signal pathway. Glycogen PAS staining demonstrated that Sirt1 OE promoted WB-F344 cells to differentiate into mature hepatocytes with glycogen synthesis ability, while Sirt1 inhibitor EX527 or Wnt/β-catenin pathway inhibitor HF535 decreased glycogen positive cells. Together, our data suggested that Sirt1 plays a vital role in activating HPCs to repair fatty liver injury or promote liver regeneration through the Wnt/β-catenin signal pathway in NAFLD, which might provide a new strategy for fatty liver injury or NAFLD therapy.

## Introduction

The liver is a hub of material metabolism in humans and animals and an important digestive and detoxification organ. However, the liver is the main target organ attacked by various adverse factors. Non-alcoholic fatty liver disease (NAFLD) is the general name of a series of diseases caused by liver fat accumulation.It starts from liver fat accumulation and then develops into steatohepatitis (NASH), cirrhosis, and even hepatic cancer (HCC) (1). In recent years, NAFLD has developed into the world's largest chronic epidemic and affected almost a quarter of the world's population (2, 3). Liver transplantation caused by NAFLD is also increasing year by year in the world. It is estimated that the number of patients with NASH and HCC will increase to 168% and 137% by 2030 (4). Presently the only treatment for NASH and HCC is liver transplantation, and there is an alarming increase in the number of patients with liver transplantation (5). However, the supply of donor organs is far from meeting the demand, resulting in the death of many patients (4). Also, with the increasing prevalence of obesity, diabetes and metabolic syndrome, the rising prevalence of NAFLD will seriously threaten human and animal health and even life (6). The liver is a metabolic organ with a strong ability of restoration and regeneration after injury. Thus, it is meaningful and urgent to explore the repair and mechanism of fatty liver injury for finding a new way to treat chronic liver disease.

The liver is a unique organ with a powerful regenerative capacity. When the liver receives partial hepatectomy or some chemical liver injury without damaging the remaining hepatocytes or other hepatocytes, liver regeneration can be achieved by residual hepatocyte proliferation. In this pattern, mature hepatocytes enter the cell cycle within 20–24 h and begin to restore the liver volume and function by mitosis (7). However, during persistent or severe liver injury, such as submassive necrosis, chronic viral hepatitis, and non-alcoholic fatty liver disease, this efficient renewal from residual hepatocyte is inhibited (8). In this scenario, hepatic progenitor cells (HPCs) are activated and proliferated. HPCs can gradually extend from the portal vein area to the liver parenchyma and differentiate into mature hepatocytes and bile duct cells to restore the damaged liver (9). In NAFLD, hepatic fat accumulation is a chronic process of liver damage, moreover, hepatic steatosis causes hepatocytes oxidative stress, which directly induces p21 expression or triggers apoptosis cascade, resulting in hepatocyte dysfunction without response to injury (10). Thus, we suppose that fatty liver injury in NAFLD can depend on the second pattern that activates HPCs to restore liver function.

HPCs (also named oval cells in rodents) have the potential capacity of bidirectional differentiation between hepatocytes and cholangiocytes and are important cell sources for the regeneration of mature hepatocytes and cholangiocytes (11). The study shows that some chronic pathological situations, such as NAFLD, chronic viral hepatitis, and alcoholic liver disease, promote the proliferation and differentiation of HPCs around the portal vein, which is called a “biliary reaction” (DR) (12). DR is the repair reaction of hepatobiliary cell injury, and HPCs, as a key component of DR, proliferates and differentiates in activated niches to regenerate damaged livers (13). The activation of HPCs can be detected through some markers, such as SOX9, EpCAM, CD133, and LGR5 (14). Studies suggest that Wnt/β-catenin signaling promotes the differentiation of HPCs into mature hepatocytes (15). Moreover, Wnt/β-catenin signaling is essential for hepatocyte proliferation, embryonic development, liver development, and maintenance of liver homeostasis (16, 17). On the contrary, the inhibition of the Wnt/ β-catenin signaling pathway impairs the differentiation of HPCs into mature hepatocytes (18).

Sirt1 is a highly conserved NAD^+^-dependent deacetylase involved in a variety of biological functions, including transcriptional silencing, cell proliferation and differentiation, senescence, apoptosis, glucose/lipid metabolism, stress response, and insulin secretion (19). Sirt1 is also very important for the activation and self-renewal of a variety of stem cells (20). The studies show that Sirt1 absence causes the senescence of hematopoietic stem cells, while Sirt1 overexpression promotes cell proliferation and cell cycle progression of mesenchymal stem cells, spermatogonial stem cells, skeletal muscle stem cells, and neural progenitor cells (21–23). Sirt1 is reported as a key factor to regulate and determine the Wnt signal pathway and cell fate. For example, Sirt1 prevents adipogenesis of bone marrow mesenchymal stem cells through deacetylating β-catenin (24). In porcine pancreatic stem cells, resveratrol-activating Sirt1 can reduce the acetylation level of β-catenin and inhibit its degradation, and thereby induce the transcriptional activation of downstream target genes related to proliferation, apoptosis, and differentiation (25).

The effect of Sirt1 on HPCs in fatty liver injury is not reported, thus, we hypothesize presently that Sirt1 plays a key role in liver repair and regeneration mediated by HPCs in the NAFLD model with liver fatty injury. Our study attempts to provide new details for revealing the activation and regulation mechanism of HPCs in chronic NAFLD.

## Materials and Methods

### Materials

Dulbecco's modified Eagle's medium (DMEM), Lipofectamine 2000, and MitoTracker Green FM reagent were purchased from GIBCO/Invitrogen (Carlsbad, CA, USA), TRIzol reagent, all restriction endonucleases, RNase-Free DNase, and M-MLV Reverse Transcriptase were purchased from TaKaRa (Dalian, China). Antibodies against SOX9, CD133, and HNF4α were purchased from Abcam (Cambridge, MA, USA). Antibodies against GSK3β, GSK3β^Tyr216^, SIRT1, and CyclinD1 were purchased from Santa Cruz Biotechnology (Santa Cruz, CA). Antibodies against β-catenin,HNF4α (p65) and the secondary antibody Alexa Fluor 488 conjugate anti-rabbit IgG, Alexa Fluor 647 conjugate anti-mouse IgG were purchased from Cell Signaling Technology (Danvers, MA, USA). Antibodies against GAPDH,LGR5, CD44, CK19, EpCAM, and HRP-conjugated secondary antibodies were from Proteintech Group (Wuhan, China). Chemiluminescence kits were purchased from Amersham Pharmacia Biotech (Piscataway, NJ, USA). The glucose assay kit and TG assay kit were purchased from Applygen Technologies Co. Ltd. (Beijing, China). Palmitic acid (PA), oleic acid (OA), insulin, and Oil Red O (ORO) were purchased from Sigma-Aldrich (St. Louis, MO, USA). Resveratrol (RSV) and Nicotinamide (NAM) were purchased from MCE Co. Ltd. (NJ, USA). Pierce Bicinchoninic Acid (BCA) protein quantitative assay kits and Nuclear and Cytoplasmic Extraction Reagent kits were purchased from ThermoFisher Scientific (Waltham, MA, USA), and a plasmid extraction kit was purchased from Tiangen Biotech Co. Ltd. (Beijing, China). Hepa1-6, WB-F344 cells were preserved in our laboratory.

### Animal Experiments

C57BL/6 background male heterozygous knockout (Sirt1^+/−^) mice were provided by Professor Peng Jian, who is from College of Animal Science and Technology &College of Veterinary Medicine, Huazhong Agricultural University, China. Both background information and genotype identification of Sirt1 knockout mice were consistent with those previously published (24, 26). Twenty-four wild-type (WT) female C57BL/6 mice weighing 20–21 g (9 weeks old) were purchased from the Experimental Animal Center of Hubei Province (Wuhan, China). All the mice were housed in individual plastic cages on a 12 h light/dark cycle with free access to water and food at room temperature. Half of 24 ten-week old mice were fed with a high-fat diet (HFD; 60 kcal% fat) for 20 weeks, and the other half were fed with a normal diet (ND; 10 kcal% fat). To evaluate the effect of Sirt1 on liver repair and regeneration *in vivo*, sixteen Sirt1^+/−^ female C57BL/6J mice weighing 18–20 g (8 weeks old) and sixteen littermate WT mice were fed HFD or ND and divided into 4 subgroups: WT mice fed with ND, WT mice fed with HFD, Sirt1^+/−^ mice fed with ND, Sirt1^+/−^ mice fed with HFD. After 20-week diet experiments, all the mice were fasted for 12 h and then sacrificed, and their tissues and sera were collected and immediately frozen in liquid nitrogen for further experiments. All the animal procedures were approved by the Hubei Province Committee on Laboratory Animal Care.

### HE and Oil Red O Staining

Fresh mouse liver was used to perform HE staining after paraffin sections and Oil Red O (ORO) staining after frozen sections. The staining result was photographed obtained by inverted fluorescence microscope IX73 (Olympus, Japan).

### Serum Routine Analysis

Fresh mouse serum samples were prepared and the serum levels of triglycerides (TG), total cholesterol (TC), glucose (Glu), glutamic-pyruvic transaminase (ALT) and aspartate-oxaloacetic transaminase (AST), and albumin (ALB) were detected by the completely automatic biochemical analyzer (Beckman, USA).

### Cell Culture and Transient Transfection

Hepa1-6 hepatocytes or WB-F344 stem cells were cultured at 37°C, 5%CO_2_ in DMEM containing 10% fetal bovine serum, penicillin (100 U/ml), and streptomycin (100 mg/ml). For treatment of Hepa1-6 cells with palmitic acid (PA). Transient transfection was performed by Lipofectamine 2000 according to the manufacturer's instructions when cells were grown to 80% confluence. Briefly, cells were incubated for 6 h in a serum-free medium containing plasmid DNA and Lipofectamine 2000. The transfection medium was subsequently replaced with DMEM supplemented with 10% calf bovine serum, and the cells were cultured for an additional 24 h and harvested.

### Real-Time Quantitative PCR

Total cellular RNA was extracted using Trizol (Thermo Fisher Scientific), followed by treatment with RNase-free DNaseI (Thermo Fisher Scientific) to remove genomic DNA contamination (Takara). RNA was then reverse transcribed into cDNA using Moloney murine leukemia virus reverse transcriptase. Half a microliter of the cDNA product was amplified by real-time PCR using gene-specific primers (Supplementary Table 1) to a total volume of 20 ml with SYBR Green Master Mix (Takara) on a CFX96 thermal cycler (Bio-Rad, Hercules, CA, USA). Relative gene expression was normalized to glyceraldehyde 3-phosphate dehydrogenase using the 2^−ΔΔCt^ method.

### Western Blotting

WB-F344 cells or liver tissue were lysed in buffer containing 50 mM Tris-HCl (pH 7.4), 1% NP-40, 0.25% sodium-deoxycholate,150 mM NaCl, 1 mM EDTA, 1 mM sodium orthovanadate,1 mM PMSF, and Sigma protease inhibitor. The supernatants were centrifuged at 10,000 g for 10 min after incubation for 30 min at 4°C. Protein concentrations were determined by BCA protein quantitative kit according to the manufacturer's instructions. The samples were separated by 12% SDS-PAGE and transferred to PVDF filters. The filters were blocked with Tris-buffered saline containing 0.1% Tween 20 and 5% skim milk, then incubated overnight with a primary antibody specific for β-actin and GAPDH (dilution 1:3000), SOX9, CD133, LGR5, CyclinD1, GSK3β, β-catenin, CD44, SIRT1, STAT3, HNF4α (dilution 1:1000). The bolts were imaged using MF-ChemiBIS Bio-Imaging Systems (DNR Bio Imaging Systems, Neve Yamin, Israel).

### Immunofluorescence

Liver tissues were embedded in OCT at −80°C and then cut into 6-μm sections. After antigen retrieval, the specimens were fixed with 4% paraformaldehyde and incubated with TBST solution (TBS-0.1% Tween 20) (Sigma-Aldrich) with 0.3% Triton solution (Sigma-Aldrich) for 20 min at room temperature, blocked with an antibody with TBST with 10% goat serum for 2 h at 4°C, incubated with primary antibody overnight at 4°C in TBST with 5% BSA, washed twice and incubated with secondary antibodies at room temperature for 1 h, and observed under confocal microscopy (Leica Microsystems, TCS-SP8TCS-SP8).

### WB-F344 Cell Differentiation

WB-F344 cells were cultured in 12-well plates. The cells were divided into four groups and were cultured in a DMEM medium. When the cells grew to about 80%, the four groups were corresponded respectively into empty pcDNA3.1 plasmid transfection group (Control), pcDNASirt1 plasmid transfection group (Sirt1 OE), pcDNASirt1 plasmid transfection with EX527 treatment group (Sirt1 OE + EX527), and pcDNASirt1 plasmid transfection with FH535 treatment group (Sirt1 OE + FH535). Then the cells for four groups were cultured in a differentiation medium added with Hepa1-6 conditioned medium, were induced to differentiate into hepatocytes for 72 h, and the samples were collected for the experiments.

### Glycogen PAS Staining

The differentiation of WB-F344 cells into hepatocytes was determined by glycogen PAS Staining with glycogen PAS staining kit. The staining result was photographed and obtained by inverted fluorescence microscope IX73 (Olympus, Japan).

### Statistical Analysis

For *in vivo* experiments, 8–10 mice were used in each subgroup. *In vitro* experiments were performed at least three times with similar results. Data were expressed as the means ± SEM. Statistical differences between groups were determined using ANOVA or Student's *t*-test depending on group size. *P* < 0.05 was considered as statistical significance.

## Results

### NAFLD Mouse Model Was Established by HFD Feeding

**Figure 1:**
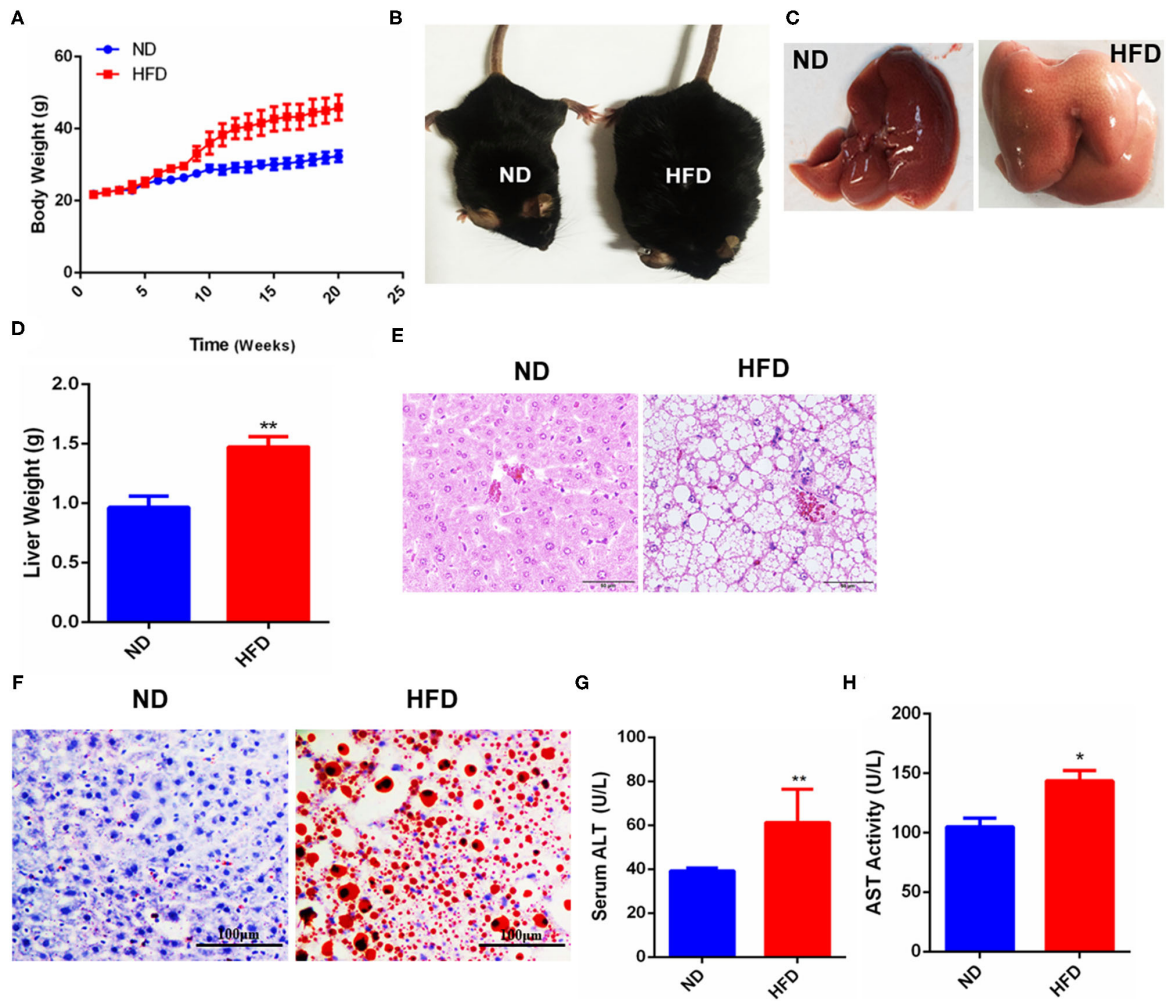
Fatty liver injury mouse model was established by HFD-feeding. **(A)** Changes of mice body weight in ND- or HFD-feeding groups. **(B)** Phenotypic difference between the control and HFD groups. **(C)** Liver morphology after dissection. **(D)** Liver weight difference between the control and HFD groups. **(E)** HE staining after paraffin sections of mouse liver. **(F)** Oil red O staining after frozen sections of mouse liver. **(G)** Analysis of serum glutamic-pyruvic transaminase (ALT) activity. **(H)** Analysis of serum aspartate-oxaloacetic transaminase (AST) activity. Data were represented by mean ± SEM (*n* = 10). *P < 0.05, **P < 0.01.

To establish the NAFLD model, mice were fed with HFD for 20 weeks as described in the methods. The results showed that body weight (BW) was increased in the mice with HFD feeding for 10 weeks later compared to the control group with ND feeding (Figure 1A). Mice phenotypic differences were observed between ND and HFD groups, and the mice of the HFD group were obese compared with those of the ND group (Figure 1B). After dissection, hepatic morphological differences were noted that the livers in the control group were dark red and smooth, and the edge was clear, while the livers in the HFD group were pale brown and swollen, and the edge was round and obtuse (Figure 1C), and the liver weight was significantly increased in the HFD group compared to the control group (Figure 1D). Liver HE staining showed that compared with the control group, the arrangement of hepatocytes in the HFD group was disordered, the cellular vacuole degeneration was severe, the structure of hepatic lobule was damaged, and the portal area and lobule were infiltrated by inflammation cells (Figure 1E). Further, Oil red O staining showed that the liver in the HFD group was accumulated by a large number of lipid droplets, while almost no lipid droplets were observed in the liver of the control group (Figure 1F). Serum analysis demonstrated that the concentration of both ALT (Figure 1G) and AST (Figure 1H) in the HFD group was significantly higher than that of the control group, suggesting liver function was seriously damaged in the HFD group. Thus, these data suggested that the mouse NAFLD model was successfully established by HFD feeding.

### HPC Proliferation Activated by Fatty Liver Injury Promoted Liver Regeneration in NAFLD

**Figure 2:**
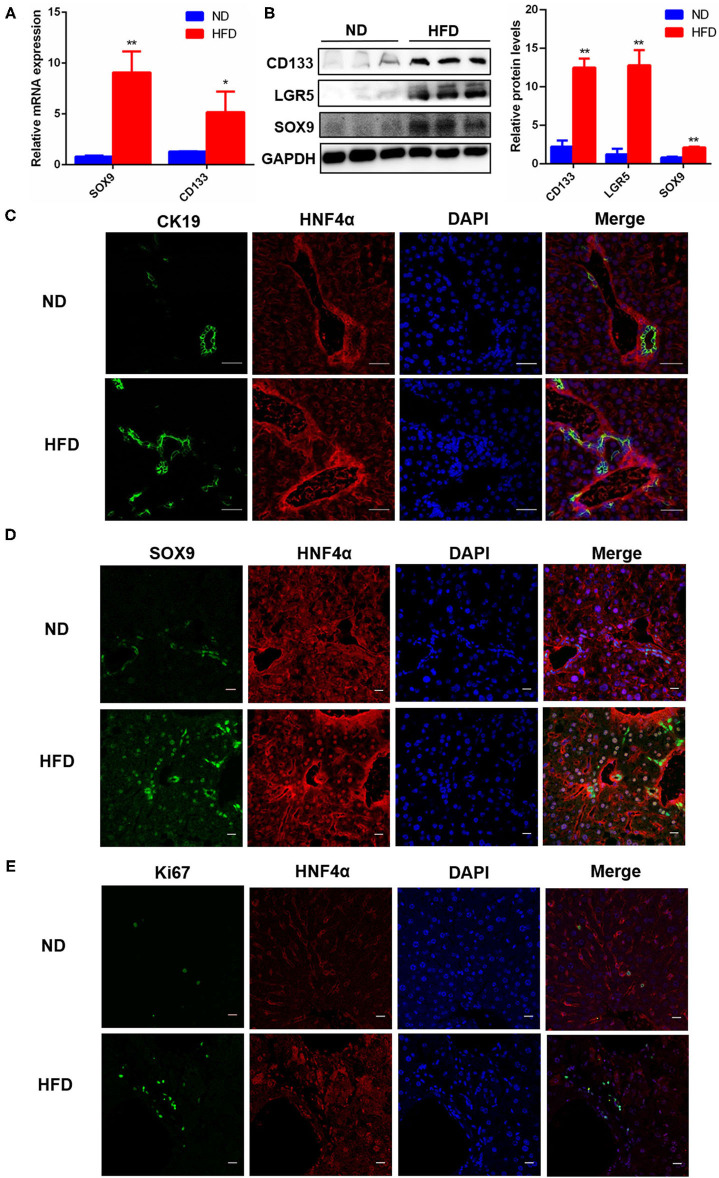
HPC proliferation was activated in fatty liver injury mouse model. **(A)** The mRNA level differences of SOX9 and CD133, two markers for HPC activation, in the liver of mice fed with ND or HFD. **(B)** The protein levels of CD133, LGR5, and SOX9 in the liver of mice fed with ND or HFD. **(C)** The proliferation of CK19^+^ cells and DR were identified by immunofluorescence staining (400×) in the liver of mice fed with ND or HFD. **(D)** The hepatic progenitor cells (HPCs) with SOX9^+^HNF4α^+^ biphenotype were identified by immunofluorescence staining (400×) in the liver of ND and HFD groups. **(E)** HPC proliferation was determined by the number of Ki67^+^ cells revealed with Immunofluorescence staining (400×) in the liver of ND and HFD groups. Data were represented by mean ± SEM (*n* = 10). *P < 0.05, **P < 0.01.

Using the above NAFLD model, we want to explore whether the repair of fatty liver damage was promoted. NAFLD is a chronic liver injury, liver repair or liver function recovery needs to depend on activating HPC proliferation, and then differentiates into mature hepatocytes and triggers liver regeneration. Therefore, we detected the expression of SOX9, CD133, and LGR5, three important markers for HPC proliferation, and the results showed that the mRNA and protein levels of *SOX9* and *CD133* were significantly increased in the fatty liver injury mice model (Figures 2A,B), and LGR5 protein level was also significantly upregulated (Figure 2B). CK19 is an important marker for bile duct cells, and the proliferation of CK19^+^ cells is thought as a sign of DR. Our immunofluorescence results revealed that CK19^+^ cells were activated and proliferated in the fatty liver injury of the mouse NAFLD model, indicating that fatty liver injury caused serious DR (Figure 2C). Furthermore, a large number of SOX9^+^HNF4α^+^ biphenotypic HPCs were widely distributed in the liver with fatty liver injury of HFD-induced NAFLD group, while in the control group, positive SOX9 was only observed in bile duct cells (Figure 2D). To determine whether activated HPCs promoted liver regeneration, Ki67^+^ cells, a key marker for liver regeneration, were examined by immunofluorescence staining, and the result showed that Ki67^+^ cells increased obviously in the liver with fatty liver injury of HFD-induced NAFLD group (Figure 2E). These data suggested that fatty liver injury in NAFLD activated HPC proliferation and promoted HPC-mediated liver regeneration.

### Fatty Liver Injury Activated Hepatic Wnt3a/β-Catenin Signaling Pathway

**Figure 3:**
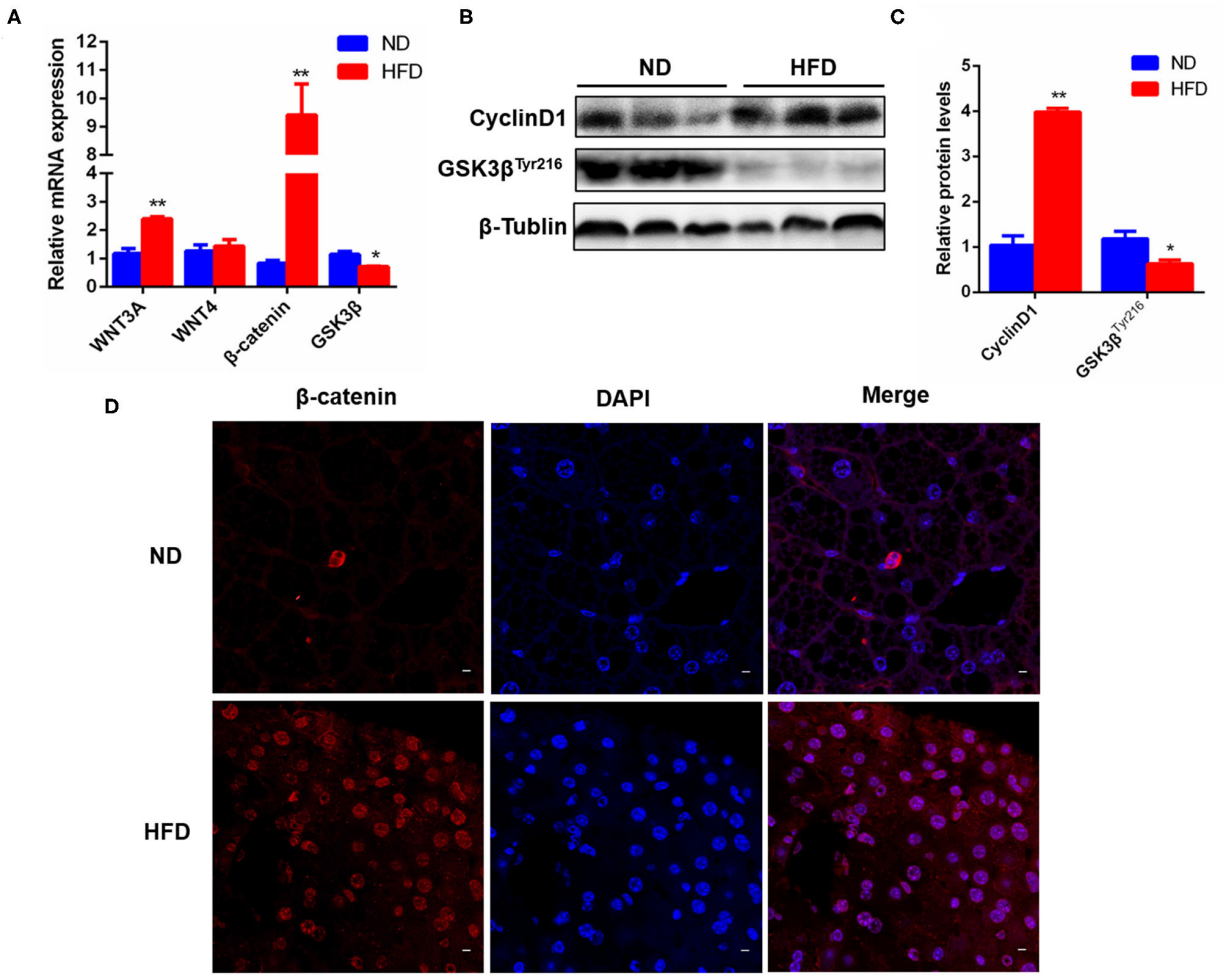
Liver fatty injury in HFD-feeding mice activated hepatic Wnt3a/β-catenin signaling pathway. **(A)** The mRNA levels of hepatic Wnt3a, Wnt4, β-catenin and GSK3 β in ND- and HFD-feeding mice. **(B)** The representative protein expression of hepatic CyclinD1 and GSK3β. **(C)** Quantitative analysis for CyclinD1 and GSK3β protein expression. **(D)** Immunofluorescence staining of β-catenin expression and translocation into the nucleus. Data were represented by mean ± SEM (*n* = 10). *P < 0.05, **P < 0.01.

Wnt/ β-catenin signal is thought as an important driving factor for liver regeneration because it is closely associated with the activation and differentiation of HPCs. To clarify that the repair of fatty liver injury in NAFLD depends on the Wnt/β-catenin signal to activate HPC proliferation and differentiation, we further detected the expression of Wnt pathway-related genes in the liver of the NAFLD model. The results showed that the mRNA levels of hepatic ligand Wnt3a (a key ligand for HPC activation in Wnt pathway), β-catenin, and their target gene CyclinD1 were significantly higher in the HFD-elicited NAFLD model than in the control, while Wnt4 (another ligand of Wnt pathway) had no significant difference. GSK3β, a negative regulator of the Wnt pathway, was significantly decreased in the liver of the HFD-induced NAFLD model (Figure 3A). The same results were acquired in the protein levels of CyclinD1 and GSK3β (Figures 3B,C). Further, the immunofluorescence staining results showed that the expression and nuclear localization of β-catenin were significantly increased in the HFD group (Figure 3D). These data revealed that the hepatic Wnt3a/β-catenin pathway was activated by liver fatty injury in HFD-feeding mice. Therefore, combined with 3.2 results, we could obtain a conclusion that liver fatty injury promoted the activation and proliferation of HPCs via the Wnt3a/ β-catenin pathway, which was conducive to the repair of liver injury.

### Sirt1 Knockdown Aggravated HFD-Elicited High Blood Glucose/Lipid and Liver Fatty Injury

**Figure 4:**
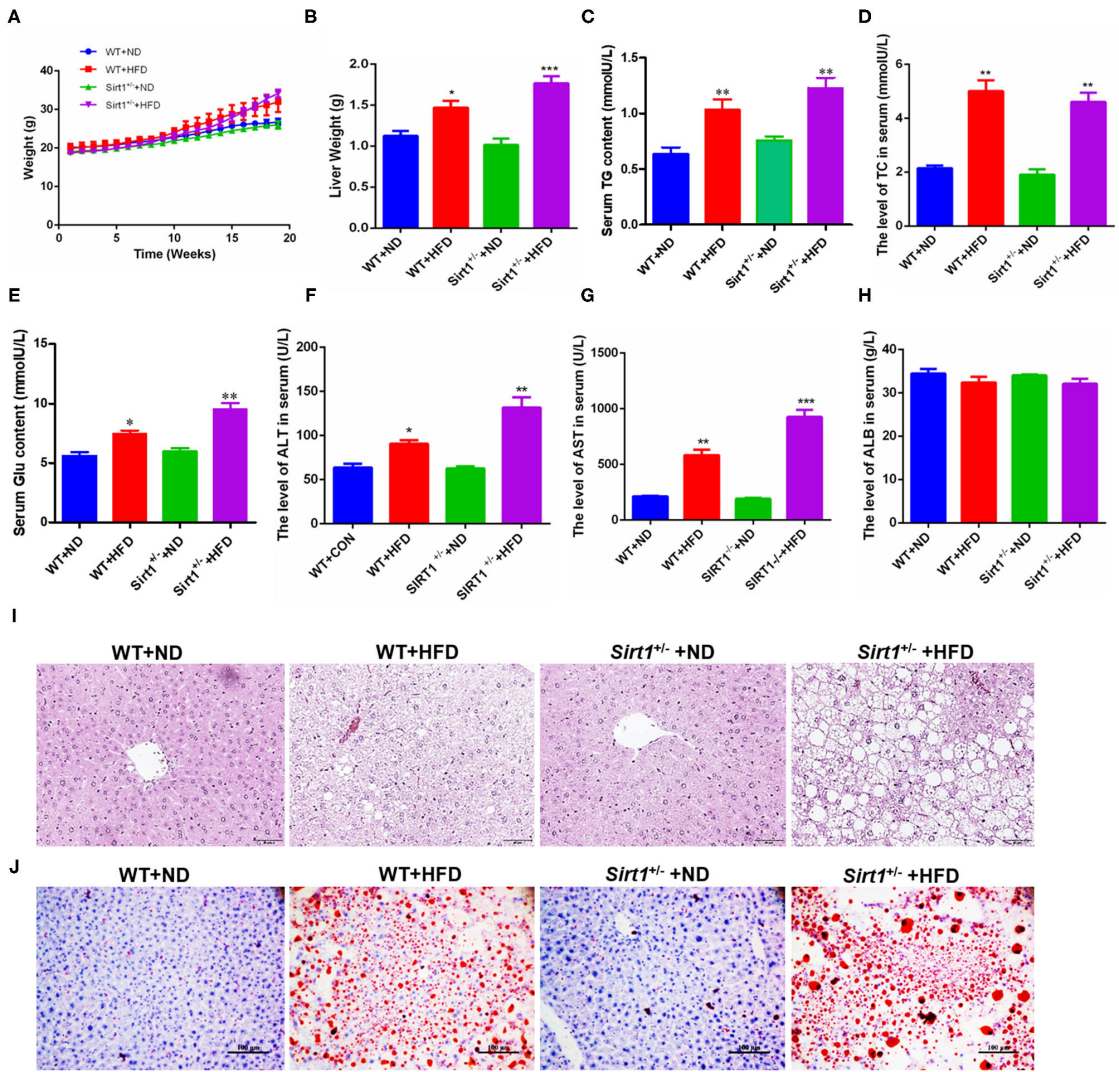
HFD-elicited liver fatty injury was aggravated in Sirt1^+/−^ mice. **(A)** The body weight change differences of mice among different groups during inducing fatty liver damage model. **(B)** Mouse liver weight after dissection. **(C–G)** The blood routine analysis for mouse serum TG, TC, glucose, ALT, AST, and ALB concentration or activity. **(I)** HE staining after paraffin section for mouse liver, 400×. **(J)** Oil red O staining after frozen section for mouse liver, 200×. **(B–H)** Data were represented by mean ± SEM (*n* = 6). *P < 0.05, **P < 0.01, ***p < 0.001.

According to all the above data, HPCs promoted the restoration of liver fatty injury via the Wnt3a/ β-catenin pathway. Also, Han et al. have reported that Sirt1 plays an important role in the maintenance, activation, and differentiation of various stem cells and adult hepatocytes (20). Thus, we were to explore the effects of Sirt1 during liver fatty injury since the effect of Sirt1 on HPC activation has not been reported presently. Our current data showed that in different subgroups, Sirt1^+/−^ mice with HFD feeding (Sirt1^+/−^ + HFD) gained the biggest weight after feeding 18 weeks, followed by wild type with HFD feeding (WT + HFD), while the body weights of wild type with ND feeding (WT + ND) and Sirt1^+/−^ with ND feeding (Sirt1^+/−^ + ND) were significantly lower in the last few weeks (Figure 4A). The liver weights of wild-type HFD and Sirt1^+/−^ HFD groups were also significantly higher than those of two corresponding ND groups and the liver weights of the Sirt1^+/−^ HFD group were higher than those of the wild-type HFD group (Figure 4B). Serum concentrations of Triglyceride (TG) (Figure 4C), total cholesterol (TC) (Figure 4D), glucose (Glu) (Figure 4E), ALT (Figure 4F), and AST (Figure 4G) in four groups showed a consistent trend, while serum albumin (ALB) had no significant effect (Figure 4H). HE staining showed that hepatocytes in wild-type ND and Sirt1^+/−^ ND groups were orderly and hepatic lobule structure was clear, while hepatocytes in Sirt1^+/−^ HFD and wild-type HFD groups were disordered, with severe cell vacuolar degeneration, hepatic lobule structure destruction, and portal area and inflammatory cell infiltration in the lobule. Notably, the Sirt1^+/−^ HFD group is more serious (Figure 4I). The results of oil red O staining showed that larger lipid droplets accumulated in the liver of the Sirt1^+/−^ HFD group than wild-type HFD group, but almost no lipid droplets were found in wild-type ND and Sirt1^+/−^ ND groups (Figure 4J). These results suggested that Sirt1 knockdown aggravated HFD-elicited liver fatty injury.

### Sirt1 Deficiency Repressed the HPC-Mediated Restoration of Liver Fatty Injury

**Figure 5:**
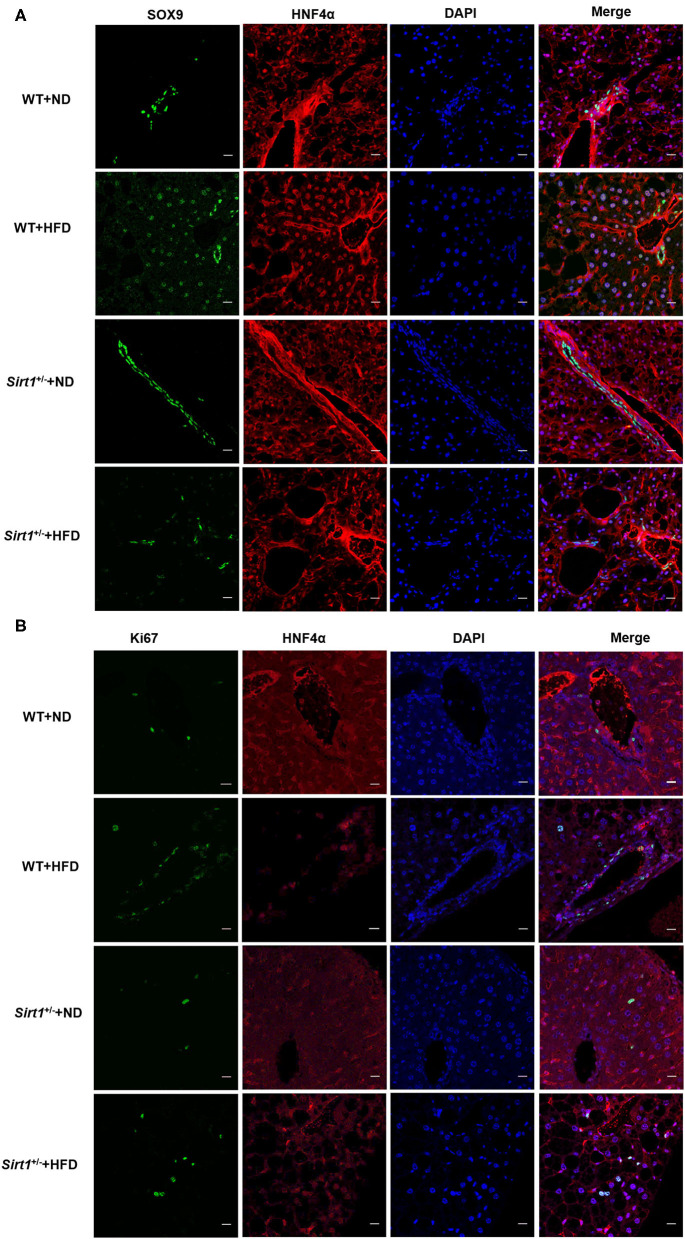
HPC-mediated restoration of liver fatty injury was inhibited in Sirt1^+/−^ mice (*n* = 6). **(A)** The hepatic progenitor cells with SOX9^+^HNF4α^+^ biphenotype were detected by immunofluorescence staining. **(B)** The Ki67^+^ cells were identified by immunofluorescence staining.

Our above data have demonstrated that liver fatty injury promoted the activation and proliferation of HPCs. Consistent with the above results, a large number of SOX9^+^ HNF4α^+^ biphenotypic HPCs appeared again in the wild-type HFD group. However, we observed that the number of SOX9^+^HNF4α^+^ biphenotypic HPCs was significantly decreased in the Sirt1^+/−^ HFD group compared to the wild-type HFD group (Figure 5A), suggesting Sirt1 knockdown inhibited HPC activation. Moreover, the number of Ki67^+^ cells in the liver of the wild-type HFD group was higher than that in the Sirt1^+/−^ HFD group, indicating that the HPC proliferation activity of Sirt1^+/−^ mice was inhibited after liver fatty injury (Figure 5B). These data suggested that HPC-mediated restoration of liver fatty injury was restrained in Sirt1^+/−^ mice.

### Effects of Palmitic Acid Stimulation on the Expression of Stemness Genes and Pathway

**Figure 6:**
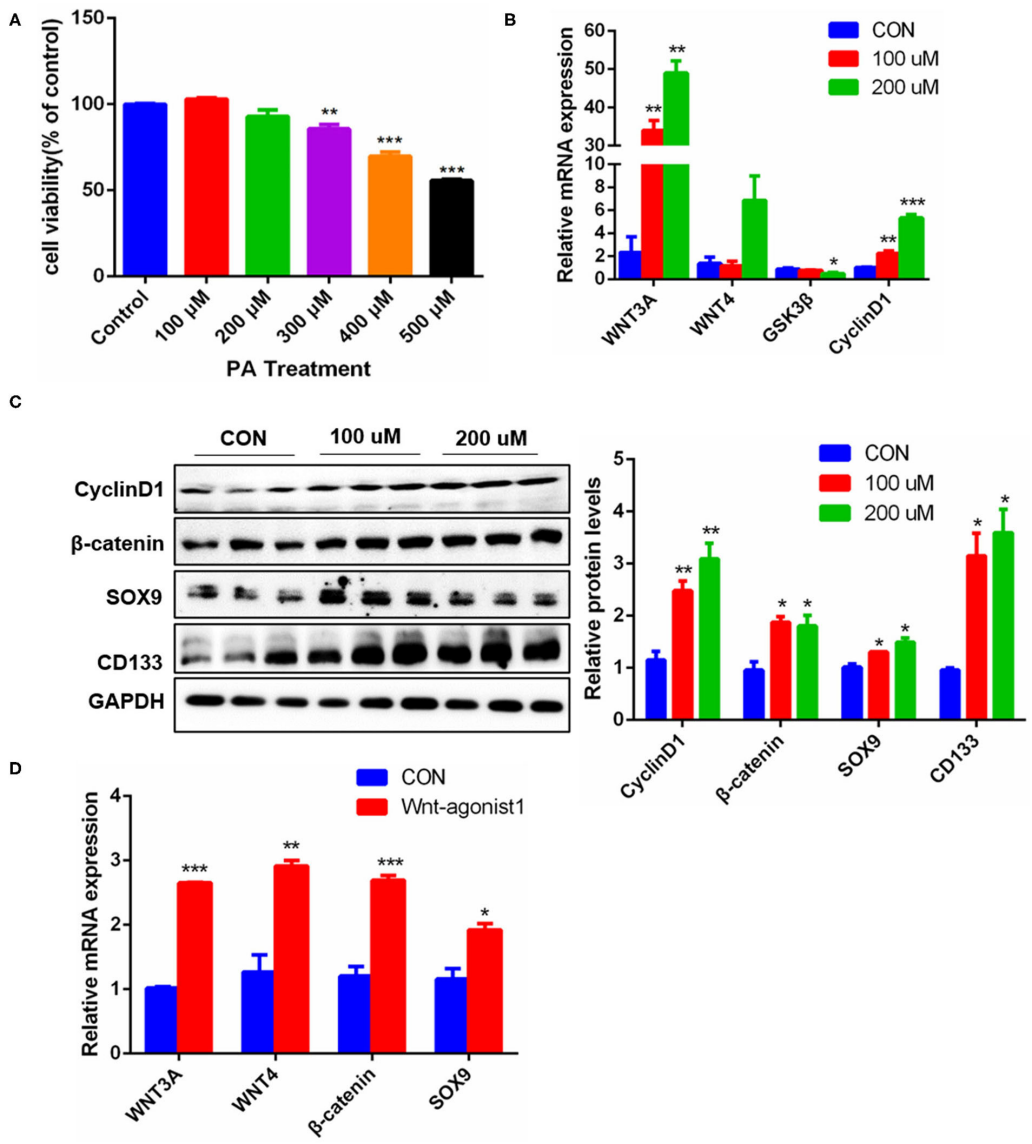
Effects of PA stimulation on liver regeneration in hepatocytes. **(A)** Effect of different PA concentrations on the viability of Hepa1-6 cells. **(B)** mRNA levels of *Wnt3a, Wnt4, GSK3*β and *CyclinD1* in Hepa1-6 cells stimulated by PA. **(C)** Protein levels of CyclinD1, β-catenin, SOX9, and CD133 in Hepa1-6 cells stimulated by PA for 24 h. **(D)** Protein levels of Wnt3a, Wnt4, β-catenin, and SOX9 in Hepa1-6 cells stimulated by Wnt agonist1 of 10uM/L for 24 h. Data were represented by mean ± SEM (*n* = 3). *P < 0.05, **P < 0.01, ***p < 0.001.

The occurrence and development of NAFLD are closely related to the excessive free fatty acids in blood circulation, especially saturated fatty acids, of which palmitic acid (PA) is an important component (27). Excessive PA can cause oxidative stress and steatosis of hepatocytes. In order to identify the effects of palmitic acid on hepatocyte proliferation, we used PA to stimulate Hepa1-6 cells. Considering the concentration of PA above 300uM had a significant effect on the survival of Hepa1-6 cells (Figure 6A), thus, we chose the PA concentration of 0uM, 100uM, and 200uM to carry on the present experiments. The results showed that After PA stimulation for 24 h, the mRNA levels of Wnt3a and CyclinD1 were increased significantly, while Wnt4 did not be affected, but GSK3β was significantly decreased (Figure 6B). Consistently, the protein expression levels of SOX9, CD133, β-catenin, and CyclinD1 were markedly increased (Figure 6C). Furthermore, Wnt-Agonist1, an activator of the Wnt pathway also upregulated the mRNA levels of SOX9, β-catenin, Wnt3a, and Wnt4 (Figure 6D). These data indicated that excessive free fatty acids damaged hepatocytes, but simultaneously promoted hepatocyte proliferation via the Wnt/β-catenin pathway, which might be conducive to the initiation of the cell repair mechanism.

### Sirt1 OE Promoted the Activation and Differentiation of WB-F344 Oval Cells

**Figure 7:**
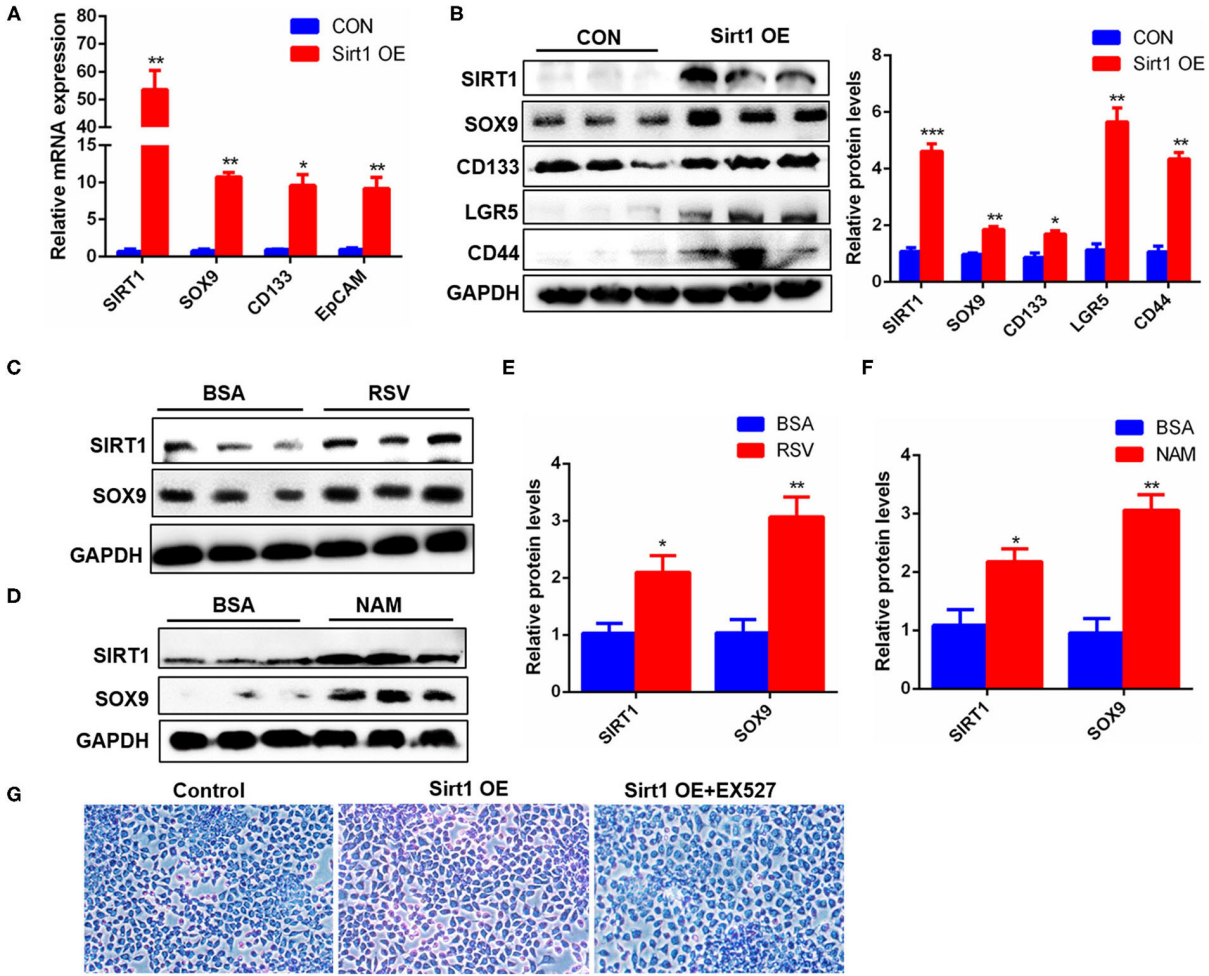
Sirt1 OE promoted the activation and differentiation of WB-F344 oval cells. **(A)** The mRNA levels of *SIRT1, SOX9, CD133*, and *EpCAM* marker genes for HPC activation in WB-F344 cells with Sirt1 overexpression. **(B)** The protein expression and quantitative analysis of SOX9, CD133, LGR5, and CD44 markers for HPC differentiation in WB-F344 cells with Sirt1 overexpression. **(C–F)** The protein expressions of SIRT1 and SOX9 in WB-F344 cells with resveratrol or nicotinamide treatment for 24 h. **(G)** Glycogen PAS Staining to determine WB-F344 cell differentiation. Data were represented by mean ± SEM (*n* = 3). *P < 0.05, **P < 0.01, ***p < 0.001.

HPCs are also called oval cells in rodents, and the WB-F344 cell line is a rat-derived oval cell line. To confirm the effect of Sirt1 on HPC activation, Sirt1 plasmids were transfected into WB-F344 cells, and the results showed that the transcriptional levels of *SOX9, CD133*, and *EpCAM* marker genes for HPC activation were significantly upregulated after Sirt1 OE (Figure 7A). Sirt1 OE also increased significantly the protein levels of HPC differentiation markers SOX9, CD133, LGR5, and CD44 in WB-F344 cells (Figure 7B). Similarly, resveratrol (RSV) and nicotinamide (NAM), two important activators for Sirt1, produced the consistent influence trend on HPC differentiation marker SOX9 with Sirt1 OE (Figures 7C–F). The hallmark function of mature hepatocytes is the ability to synthesize glycogen. Therefore, we determined whether WB-F344 hepatic progenitor cells differentiated into mature hepatocytes by glycogen PAS staining. And the results showed that there were more glycogen positive cells in the Sirt1 OE group compared to control, but there was almost no glycogen positive cells in the Sirt1 OE group with Sirt1 inhibitor EX527 treatment (Sirt1 OE + EX527) (Figure 7G). These data revealed that Sirt1 OE promoted the activation and differentiation of WB-F344 hepatic progenitor cells.

### Sirt1 OE Promoted the Activation and Differentiation of WB-F344 Cells Through the Wnt/β-catenin Pathway

**Figure 8:**
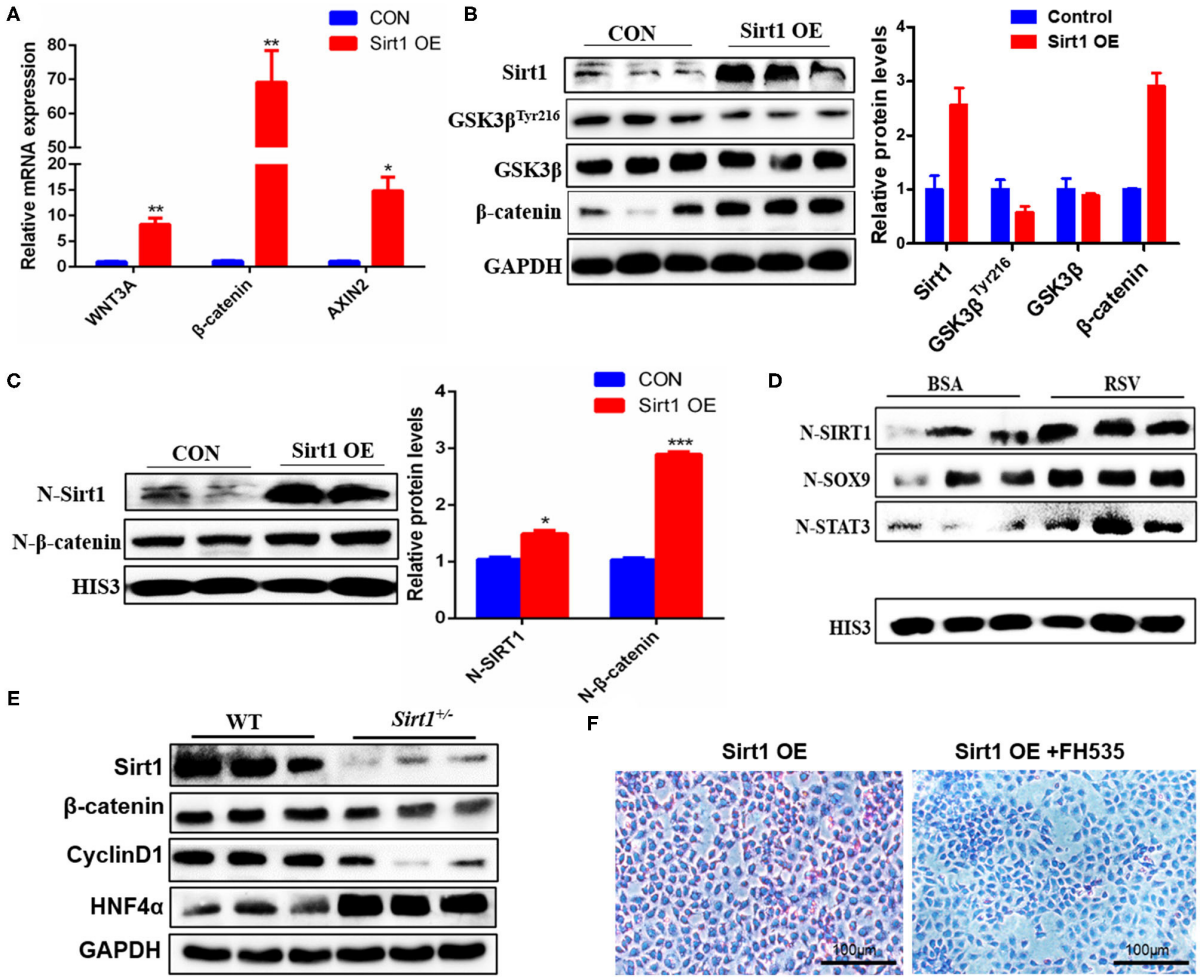
Sirt1 promoted the activation and differentiation of WB-F344 cells through the Wnt/β-catenin pathway. **(A)** The mRNA levels of Wnt3a, β-catenin and AXIN2 in Sirt1-overexpressed WB-F344 cells. **(B)** The protein expressions of SIRT1, GSK3β^Tyr216^, GSK3β and β-catenin in Sirt1-overexpressed WB-F344 cells. **(C)** The nuclear entry level of Sirt1 and β-catenin in Sirt1-overexpressed WB-F344 cells. **(D)** The nuclear protein was extracted from WB-F344 cells stimulated by resveratrol for 24 h and the levels of nuclear SIRT1, STAT3, SOX9, and β-catenin were detected by Western Blot. **(E)** The protein expressions of SIRT1, β-catenin, CyclinD1 and HNF 4a in the liver of Sirt1^+/−^ mice were detected by Western Blot. **(F)** Glycogen PAS Staining to determine WB-F344 cell differentiation. Data were represented by mean ± SEM (*n* = 3). *P < 0.05, **P < 0.01, ***p < 0.001.

To clarify the mechanism of Sirt1 regulating HPC differentiation, we analyzed the effect of Sirt1 OE on the Wnt/β-catenin signal pathway. The present results revealed that overexpressed Sirt1 significantly increased the mRNA levels of *Wnt3a*, β*-catenin*, and *AXIN2* in WB-F344 oval cells (Figure 8A). GSK3β is a repressor of Wnt3a/β-catenin pathway, and GSK3β-Tyr216 phosphorylation/activation promotes β-catenin degradation. Our Western Blot results showed that Sirt1 OE inhibited significantly the GSK3β-Tyr216 phosphorylation (Figure 8B) and increased the levels of total and nuclear β-catenin (Figures 8B,C). STAT3, SOX9, and β-catenin are important transcription factors determining HPC proliferation and differentiation. After WB-F344 cells were stimulated by Sirt1 activator resveratrol (RSV) for 24 h, the nuclear protein was extracted from WB-F344 cells and the levels of nuclear SirT1, STAT3, SOX9, and β-catenin were detected by Western Blot. The results showed that RSV enhanced the entry of Sirt1, STAT3, SOX9, and β-catenin into the nucleus (Figure 8D), which is in line with the conclusion *in vivo* that Sirt1 knockdown repressed the expression of β-catenin and CyclinD1, and enhanced the expression of HFN4α (Figure 8E), suggesting inhibiting HPC proliferation and differentiation. To understand fully the regulation of Sirt1 on HPC differentiation via Wnt/β-catenin, after Sirt1 plasmid was transfected into WB-F344 cells for 24h, we used FH535, an effective inhibitor of the Wnt/β-catenin pathway, to treat the cells, then induced the cells to differentiate into hepatocytes for 72 h. Glycogen PAS staining results showed that glycogen positive cells were markedly decreased in Sirt1 OE with the FH535 treatment group (Sirt1 OE+FH535) compared to the Sirt1 OE group (Figure 8F), suggesting that the differentiation of HPCs into hepatocytes activated by Sirt1 depends on the Wnt/β catenin pathway.

## Discussion

The liver is an important metabolic organ of the body, which has a strong ability to regenerate after injury (9). With the increase in the incidence of fatty liver disease, the aplastic disorder has become an important clinical problem (28). NAFLD is the main cause of chronic liver disease in many parts of the world, and fat is the most common cause of non-alcoholic steatohepatitis. In the present study, we chose HFD-elicited NAFLD mice as a chronic liver injury model to explore HPC-mediated fatty liver injury and repair as well as the corresponding mechanism.

After 20 weeks of HFD induction, mice presented obese phenotypes, and the liver was accumulated a large amount of fat and infiltrated a lot of inflammatory cells (Figures 1A–F), suggesting that HFD caused mice fatty liver or lipid toxicity and liver fat accumulation elicited the recruitment of inflammatory cells and further developed into NAFLD. And the significant increase of ALT and AST concentrations in serum also indicates that liver function has been seriously damaged (Figures 1G,H). Clinically, DR is often observed in patients with chronic liver disease, and most of the hepatocytes in these patients have proliferation disorders (29). It was previously reported that the CK19 positive area was enlarged in liver tissues of patients with NAFLD, which represented the occurrence of bile duct reaction. In addition, bile duct reaction was higher in NASH patients with severe liver fibrosis, suggesting that bile duct reaction was associated with the progression of non-alcoholic steatohepatitis (30). In our liver fat injury model, obvious DR was observed, CK19 positive cells significantly increased, and bile duct cells spread and presented atypical morphology (Figure 2C). This phenomenon indicated that hepatic progenitor cells (HPCs) might originate from bile duct cells rather than hepatocytes. In non-alcoholic fatty liver, oxidative stress plays a major role in hepatocyte replication disorder by directly inducing p21 expression or by triggering apoptosis cascade (31). The damage of hepatocyte regeneration and the increase of hepatocyte injury caused by long-term oxidative stress are the common results of HPC activation and differentiation (30). In the liver fatty injury model at present, hepatic SOX9^+^HNF4α^+^ biphenotype suggested that HPCs were activated by fatty liver injury (Figure 2D). SOX9 is a member of the Sry-related high mobility family box transcription factor, which plays a key role in the embryonic formation of many tissues and organs, including chondrocytes, testis, heart, lung, pancreas, bile duct, hair follicles, retina, and central nervous system (14). It has been reported that hybrid hepatocytes expressing SOX9 and HNF4 α have been identified as HPCs in Herring tube, and they proliferate vigorously and then differentiate into hepatocytes after liver injury (32–34). In our study, liver fatty damage also caused the activation of HPCs expressing SOX9 and HNF4 α, which were also enriched near the Herring tube. The increased expression of SOX9, CD133 and LGR5 (markers for HPC proliferation) (Figures 2A,B), DR with CK19^+^ phenotype (Figure 2C) as well as Ki67 positive phenotype (Figure 2E) also demonstrated the activation HPC proliferation in liver fatty damage.

Many studies have reported that Wnt/ β-catenin signal pathway is an important driver of liver injury repair and regeneration and plays a key role in the activation, proliferation, and differentiation of adult liver progenitor cells (18, 35). Our experimental results show that either mouse liver fat injury or PA stimulation of Hepa1-6 cells enhanced the Wnt/β-catenin signal (Figures 3 and 6). In the previous report, Wnt3a can stimulate the proliferation activity of HPCs *in vitro*, and Wnt/ β-catenin signal pathway is activated obviously in HPC proliferation induced by DDC diet in mice (36). Our present study found that in liver fat injury, the activation of the Wnt pathway stimulated HPC proliferation (Figures 1–3). Liver fat injury caused the upregulation of Wnt3a expression (Figure 3A) and the downregulation of GSK3β expression (Figures 3B,C), a repressor of Wnt3a/β-catenin pathway, which stimulated the entry of β-catenin into the nucleus (Figure 3D). This increase in nuclear β-catenin promoted the expression of its target gene CyclinD1 (Figure 3B). CyclinD1 is a cell cycle key regulator to promote the process of the G1 cell cycle to the S phase which is a marker of cell proliferation. The other study shows that the proliferative activity of rat oval cells is increased significantly in AAF/PHx model, and the nuclear β-catenin staining was positive (16). Therefore, the activation of the Wnt/β-catenin pathway might be indispensable for promoting HPC proliferation in the repair of liver injury and liver regeneration.

It has been demonstrated that Sirt1 affects the maintenance, activation, and senescence of many kinds of stem cells (19, 37). Here our results showed that HFD-elicited liver fatty injury was seriously aggravated in Sirt1^+/−^ mice (Figure 4), and Sirt1 knockdown significantly inhibited the activation of HPCs according to the number of SOX9^+^HNF4α^+^ biphenotypic HPCs as well as the proliferation of HPCs based on Ki67^+^ cells in the liver of Sirt1^+/−^ mice with HFD feeding (Figure 5). Reversely, *in vitro* Sirt1 OE or activation with Sirt1 activators (RSV or NAM) significantly upregulated the markers of HPCs in rat-derived WB-F344 oval cells (Figure 7). These data indicated that Sirt1 had a positive effect on the activation of HPCs. It is worth noting that the role of NAM in regulating Sirt1 activation is still controversial. NAM is generally considered to be the product of deacetylation catalyzed by Sirt1 and is considered to have product feedback inhibition on Sirt1 activity. However, in the present study, we found that NAM is a Sirt1 activator and promoted its expression and function in cell proliferation (Figures 7D,F). The possible reason is that NAM is the main precursor of intracellular NAD^+^ synthesis through the salvage pathway, while NAD^+^ can promote Sirt1 activation. The previous study of Jang et al. (38) also obtained a similar result that supplementation of NAM in cell culture increased the level of intracellular NAD^+^ and lead to the activation of Sirt1.

LGR5 is a Wnt target gene with low expression in the normal liver and also a receptor of Wnt agonist R-spondin1 (39). Huch et al. found that LGR5^+^ cells appeared near the bile duct after liver injury in mice, and the LGR5^+^ cells differentiated into BECs and hepatocytes (15). In our study, we also found that Sirt1 OE increased significantly the expression of LGR5 (Figure 7B). Thus, Sirt1 might regulate the Wnt signal pathway by stimulating the expression of Wnt target gene LGR5 and thereby promoting liver regeneration. STAT3 plays a key role in liver regeneration, promoting hepatocyte proliferation in hepatocyte-mediated liver regeneration and initiating cell progression from the G1 phase to the S phase (40). As an important transcription factor, SOX9 can participate in a variety of genetic pathways, regulate stem cell self-renewal ability and multi-directional differentiation potential, and promote the development of stem cell and progenitor cell niches in tissues and organs (41). Indeed, we found that activating Sirt1 increased the expression of SOX9, a marker for HPC differentiation (Figures 7C,D) and the entry of SOX9 and STAT3 into the nucleus (Figures 8C,D) and increased glycogen positive cells (Figure 8F), indicating that more HPCs differentiated into mature hepatocytes.

It is reported that endogenous or exogenous activation of β-catenin improves liver regeneration in animal models and patients (42). Our current results showed that Sirt1 OE in WB-F344 oval cells inhibited significantly the GSK3β-Tyr216 phosphorylation (Figure 8B) and increased the levels of total and nuclear β-catenin (Figures 8B,C), suggesting that Sirt1 increased activity of Wnt/ β-catenin. On the contrary, we also proved this conclusion because Sirt1 knockdown inhibited significantly Wnt/ β-catenin pathway in the liver of Sirt1^+/−^ mice (Figure 8E). Further, glycogen PAS staining results showed that glycogen positive cells were markedly decreased in Sirt1 OE after β-catenin inhibitor FH535 treatment compared to the Sirt1 OE group (Figure 8F). These data suggested that Sirt1-activating the differentiation of HPCs into hepatocytes need depend on the Wnt/β-catenin pathway. Actually, in bone marrow mesenchymal stem cells, Sirt1 has also been demonstrated to activate the Wnt/β-catenin pathway, and it can promote β-catenin accumulation in the nucleus by deacetylating β-catenin (24), which is consistent with our present results in hepatic progenitor cells.

**Figure 9:**
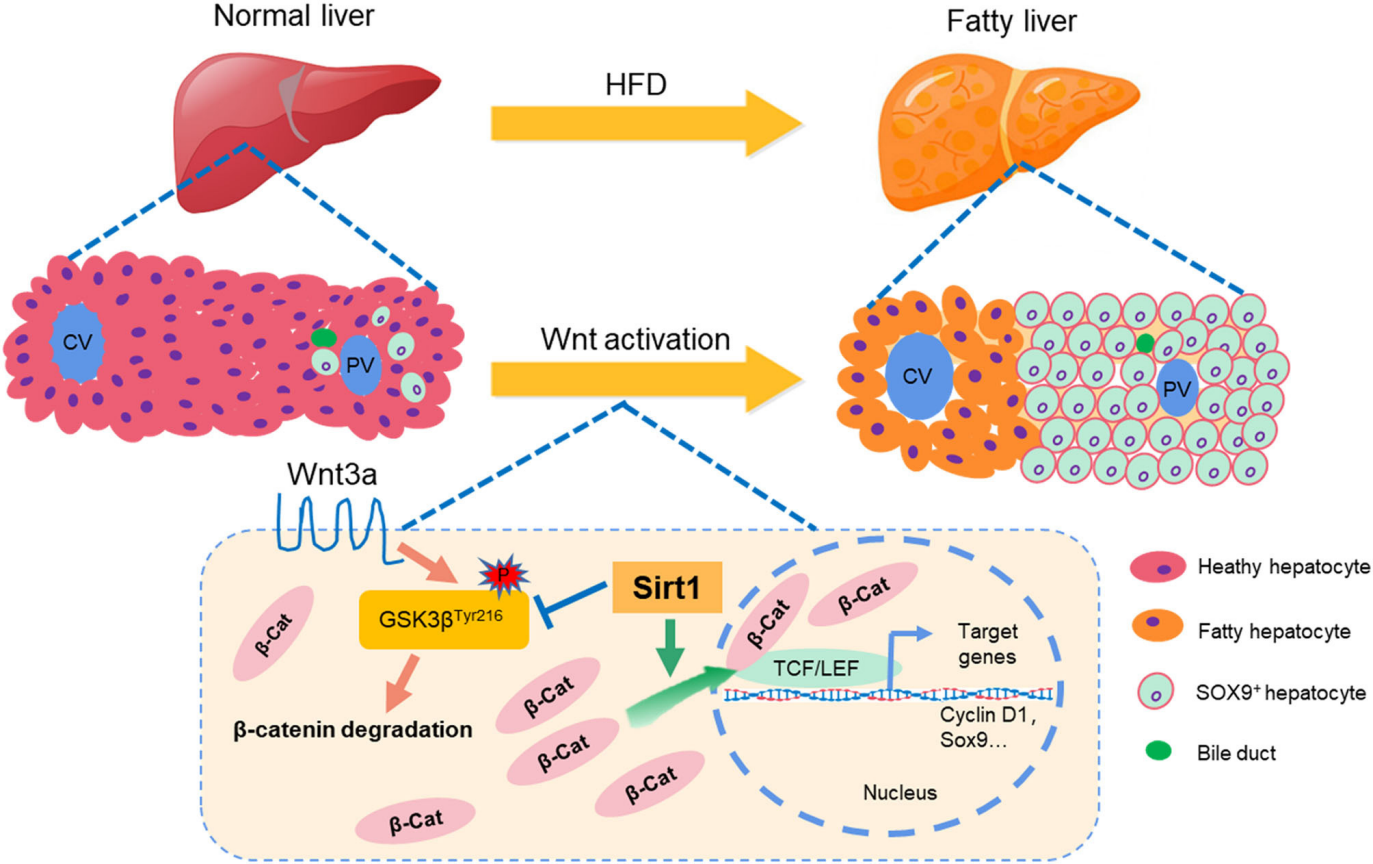
A schematic diagram of the experimental results.

In conclusion, as summarized in Figure 9, our data suggested that liver fat injury induced by HFD caused a significant biliary reaction and SOX9^+^HNF4α^+^ biphenotypic HPCs were activated in the fatty liver of mice with enhanced proliferative activity, and Sirt1 plays a vital role in activating HPCs to repair the fatty liver injury and promote liver regeneration through Wnt/β-catenin signal pathway in NAFLD, which might provide a new strategy for fatty liver injury or NAFLD therapy.

## Data Availability Statement

The original contributions presented in the study are included in the article/Supplementary Material, further inquiries can be directed to the corresponding author/s.

## Ethics Statement

The animal study was reviewed and approved by Hubei Province Committee on Laboratory Animal Care; Wuhan, Hubei, China. Written informed consent was obtained from the owners for the participation of their animals in this study.

## Author Contributions

QL and XC: conceptualization and resources. QL, YG, YW, and XC: methodology and data curation. QL, YG, YW, YC, and BL: investigation. QL, YG, YC, and XC: original draft preparation. QL, YG, and XC: review and editing, visualization, and supervision. XC: project administration and funding acquisition. All authors contributed to the article and approved the submitted version.

## Funding

This work was supported by the National Key Research and Development Program of China (2017YFA010320-01), Natural Science Foundation of China (31572382), and Fundamental Research Funds for the Central Universities (2662018PY020).

## Conflict of Interest

The authors declare that the research was conducted in the absence of any commercial or financial relationships that could be construed as a potential conflict of interest.

## Publisher's Note

All claims expressed in this article are solely those of the authors and do not necessarily represent those of their affiliated organizations, or those of the publisher, the editors and the reviewers. Any product that may be evaluated in this article, or claim that may be made by its manufacturer, is not guaranteed or endorsed by the publisher.
