# Supplementary material for: Sirt1 Promotes the Restoration of Hepatic Progenitor Cell (HPC)-Mediated Liver Fatty Injury in NAFLD Through Activating the Wnt/β-Catenin Signal Pathway
Source: Front Nutr. 2021 Dec 15;8:791861. doi: 10.3389/fnut.2021.791861 (PMC8714951; doi:10.3389/fnut.2021.791861)
Supplement: Supplementary file 1 [file Table_1.DOCX]

**Supplemental Table 1 Primer sequences for RT-PCR analyses.**

| Target | Sequence |
| --- | --- |
| Mus GAPDH Forward Primer | 5′- GGCTACCGAGACAACC-3′ |
| Mus GAPDH Reverse Primer | 5′- CCACAGCGTCATATCATC-3′^，^ |
| Mus SIRT1 Forward Primer | 5′- GGCTACCGAGACAACC-3′ |
| Mus SIRT1 Reverse Primer | 5′- CCACAGCGTCATATCATC-3′ |
| Mus WNT3A Forward Primer | 5′- CAGTGCCTCGGAGATGGTG -3′ |
| Mus WNT3A Reverse Primer | 5′- GGTTAGGTTCGCAGAAGTTGG-3′ |
| Mus AXIN2 Forward Primer | 5′- TGACTCTCCTTCCAGATCCCA-3′ |
| Mus AXIN2 Reverse Primer | 5′- TGCCCACACTAGGCTGACA-3′ |
| Mus EpCAM Forward Primer | 5′- GCGGCTCAGAGAGACTGTG -3′ |
| Mus EpCAM Reverse Primer | 5′- CCAAGCATTTAGACGCCAGTTT -3′ |
| Mus PROM1 Forward Primer | 5′-CTCCCATCAGTGGATAGAGAACT-3′ |
| Mus PROM1 Reverse Primer | 5′- ATACCCCCTTTTGACGAGGCT -3′ |
| Mus PCNA Forward Primer | 5′- TTTGAGGCACGCCTGATCC -3′ |
| Mus PCNA Reverse Primer | 5′- GGAGACGTGAGACGAGTCCAT -3′ |
| Mus CK19 Forward Primer | 5′- GGGGGTTCAGTACGCATTGG -3′ |
| Mus CK19 Reverse Primer | 5′- GAGGACGAGGTCACGAAGC -3′ |
| Mus SOX9 Forward Primer | 5′- CGGAACAGACTCACATCTCTCC-3′ |
| Mus SOX9 Reverse Primer | 5′- GCTTGCACGTCGGTTTTGG-3′ |
| Mus WNT4 Forword Primer | 5′-AGACGTGCGAGAAACTCAAAG-3′ |
| Mus WNT4 Reverse Primer | 5′- GGAACTGGTATTGGCACTCCT -3′ |
| Mus CTNNB1 Forward Primer | 5′- ATGGAGCCGGACAGAAAAGC -3′ |
| Mus CTNNB1 Reverse Primer | 5′- CTTGCCACTCAGGGAAGGA -3′ |
| Mus GSK3β Forward Primer | 5′- TGGCAGCAAGGTAACCACAG -3′ |
| Mus GSK3β Reverse Primer | 5′- CGGTTCTTAAATCGCTTGTCCTG -3′ |
